# Cardiac Genetic Variants in Sudden, Unexpected Death in Epilepsy: From Challenging DNA Extraction Methods to Updated NGS Panels for Improved Genetic Analysis

**DOI:** 10.3390/genes16111272

**Published:** 2025-10-28

**Authors:** Alessia Bernini Di Michele, Valerio Onofri, Filomena Melchionda, Lucia Fiordelmondo, Eleonora Ciarimboli, Marco Palpacelli, Sara Sablone, Chiara Turchi, Mauro Pesaresi

**Affiliations:** 1 Section of Legal Medicine, Department of Biomedical Sciences and Public Health, Polytechnic University of Marche, Via Tronto, 60126 Ancona, Italy; a.bernini@pm.univpm.it (A.B.D.M.); f.melchionda@univpm.it (F.M.); m.pesaresi@univpm.it (M.P.); 2 Legal Medicine Unit, A.O.U. Azienda Ospedaliero Universitaria delle Marche, 60126 Ancona, Italy; valerio.onofri@ospedaliriuniti.marche.it (V.O.); lucia.fiordelmondo@ospedaliriuniti.marche.it (L.F.); eleonora.ciarimboli@ospedaliriuniti.marche.it (E.C.);; 3 Section of Legal Medicine, Interdisciplinary Department of Medicine, University of Bari “Aldo Moro”-Bari Policlinico Hospital Giulio Cesare Square, 11, 70124 Bari, Italy; sara.sablone@policlinico.ba.it

**Keywords:** SUDEP, molecular autopsy, cardiac genes, next generation sequencing

## Abstract

Background/Objectives: SUDEP is the sudden, unexpected death of someone with epilepsy, and occurs mainly during sleep or at rest, or when the individual does not seem to have experienced a convulsive seizure. The cause of death in SUDEP is still unknown, and it may differ between cases. Cardiac factors are among the most prevalent causes observed in SUDEP. Therefore, within the forensic medicine framework, identifying well-known DNA markers involved in cardiac sudden and unexpected death would aid in understanding the cause of SUDEP, as well as in finding cardiac risk markers in patients with epilepsy. The purpose of this study was to identify any genetic variants by analyzing blood and formalin-fixed paraffin-embedded (FFPE) tissue samples, utilizing next-generation sequencing techniques. Methods: We investigated five cases of SUDEP that were examined at the Legal Medicine department of Ancona (Italy). Peripheral blood or FFPE cardiac tissues were collected, and different DNA extraction methods were performed. In particular, this study underlines a new extraction method from FFPE tissue, adapting the Casework kit for forensic application to our purpose. Later, about one hundred genes correlated to inherited cardiac diseases were sequenced through the Ion PGM System and Ion GeneStudio S5 Systems. Results: Bioinformatic analysis showed some genetic variants of unknown significance (VUS) on genes involved in SUDEP: RYR2, SCN8A, and AKAP9. Conclusions: As expected, very low coverage of the target base was observed for FFPE tissue samples because of the complexity of the biological material. Therefore, the presence of any significant variants in unamplified regions cannot be excluded in the FFPE samples. As suggested by the literature, the variants found in the blood samples are potentially associated with SUDEP.

## 1. Introduction

SUDEP is defined as a sudden, unexpected, witnessed or unwitnessed death in a non-traumatic or non-drowning manner, with or without evidence of a seizure—excluding documented status epilepticus—in which there is no toxicological or anatomical cause of death [1]. Each year, more than 1 in 1000 people with epilepsy die from SUDEP. Most SUDEP cases are observed in young subjects (0–15 years old), and the incidence is higher in men (1.41/1000 person-years) than in women (0.96/1000 person-years) [2]. However, it is noted that the risk of SUDEP has been underestimated, especially in older people, regardless of sex [2]. These deaths occur mainly during sleep or at rest, and the individuals affected do not seem to experience convulsive seizures. The cause of death by SUDEP is still unknown, and it may differ between cases.

The overarching term SUDEP can be subdivided into four different categories: Definite, Definite Plus, Probably, and Possible; otherwise, if an alternate cause of death has been determined, the possibility that SUDEP is the cause of death is excluded (SUDEP Unlikely).

1. Definite SUDEP: a non-traumatic and non-drowning death in an individual with epilepsy, without a cause of death after postmortem examination.
2. Definite SUDEP Plus: includes the presence of a concomitant condition other than epilepsy, where death may be due to the combined effects of both epilepsy and the other condition.
3. Probably SUDEP: all the same criteria for Definite SUDEP are met, but no postmortem examination is performed.
4. Possible SUDEP: insufficient information is available regarding the death; post-mortem examinations have not been carried out, and the SUDEP hypothesis cannot be ruled out [3,4,5].

Recent studies have extensively investigated the pathophysiology of SUDEP in different groups, proposing four main mechanisms for SUDEP: cardiac dysfunction, such as cardiac arrhythmias and other cardiac events; respiratory factors, such as seizure-induced pulmonary dysfunction; and cerebral and autonomic nervous system dysregulation. Other less frequent causes of SUDEP are anti-epileptic drugs; vagal nerve stimulation, which may induce bradycardia or cardiac arrest; and genetic factors, including mutations in several genes associated with an increased susceptibility to SUDEP [6]. Over 33% of these are related to mutations, leading to increased susceptibility to arrhythmia [7]. Cardiac factors such as channelopathies, cardiomyopathies, aortopathies, cardiac arrhythmias, and long QT syndrome (LQTS) are among the most involved elements in SUDEP. Therefore, within the forensic medicine framework, the well-known DNA markers involved in cardiac sudden and unexpected death would aid in understanding the cause of SUDEP and in finding cardiac risk markers in patients with epilepsy. The SUDEP research is supported by genetic analysis (so-called molecular autopsy) that can lead to the identification of variants with clinical significance related to the cause of death.

To date, few genes have been analyzed in genetic testing, although the implementation of next-generation sequencing (NGS) and the low cost of these high-throughput technologies are helping to identify new candidate genes. Few studies have examined possible genetic risk factors in SUDEP. One of the limiting factors is that quality DNA samples must be collected during the life of the patient or extracted from postmortem blood. Although there is increasing awareness of the importance of collecting blood samples in unexplained sudden death in order to perform post-mortem genetic testing of the main cardiac arrhythmia genes [8], it is not always suitable for laboratories [9]. As in our case, we had collected formalin-fixed paraffin-embedded (FFPE) tissue in the past years for further analyses, with no blood samples available. Bagnall et al. in 2017 [8] performed exome sequencing from degraded DNA derived from FFPE postmortem tissues, which identified a de novo *SCN1A* frameshift variant in a patient with SUDEP. This study shows the possibility of analyzing historical collections of postmortem fixed tissues from SUDEP cases [8,9]. Another limitation of studies on SUDEP is the small number of cases. In fact, it is a rare event, and the largest genetic study of SUDEP was performed by Bagnall et al. in 2016 using exome sequencing-based analysis of 61 SUDEP cases [10].

In our forensic genetics laboratory, we analyzed two SUDEP cases in 2022 from FFPE heart tissues on a cardiac custom panel of 97 genes, and, more recently, we studied three other cases, two samples of whole blood and one sample of FFPE heart tissue, through an updated custom panel of 103 genes. We used a panel of cardiac genes because SUDEP was hypothesized to be caused by cardiac factors. In this study, we included not only samples of whole blood, but we also discussed the analysis of FFPE tissues, which were the only available biological matrices for some cases. Sometimes, it is common to store FFPE tissue for further analyses, since it can be stored for a longer time at room temperature at a lower cost. For this reason, we drew attention to the semi-automated DNA extraction method used for FFPE tissue and the results that we obtained.

## 2. Materials and Methods

In our research, we included five cases of SUDEP, renamed as sample 1, sample 2, sample 3, sample 4, and sample 5. In the study, two males and three females were included. All the subjects were epileptic patients treated with anti-epileptic drugs. In particular, sample 1 was a 31-year-old male who died in his sleep; sample 2 was a 47-year-old man with epilepsy under treatment; sample 3 was a 30-year-old woman with epilepsy and in drug treatment; sample 4 corresponded to a 56-year-old woman with cardiomyopathy who was HIV-positive; and finally, the most recent case of SUDEP, sample 5, was a 23-year-old young woman found unconscious lying in an apartment. For all cases, the toxicology tests revealed negative results for alcohol and psychoactive substances, and the anti-epileptic drugs present in the subjects in quantities were compatible with a therapeutic intake. Based on these findings, the coroner determined the cause of death to be attributable to Definite SUDEP for all cases, with the exception of sample 4, who suffered from cardiomyopathy and was classified as Definite Plus SUDEP, due to the appearance of fatal arrhythmia. The starting biological material was different: samples 1, 2, and 3 were FFPE heart tissue samples, and samples 4 and 5 were whole blood samples. Extraction of DNA from FFPE tissue is a critical step in molecular genetic testing. The area of the tissue was cut with a scalpel, and then the paraffin was eliminated, leaving only the tissue on the surface. This step facilitates slicing the tissue with the microtome without the interference of paraffin in the next step of extraction. Five to ten 5 μm thick slices were cut with the microtome, and a semi-automated DNA extraction method was performed following the “Promega Corporation, Revised 5/12, part #TB382” protocol of the Casework Kit (catalog number: AS1550, Promega, Madison, WI, USA) with the Maxwell^®^ 16 Instrument (Promega). A total of 23 μL of Proteinase K solution and 180 μL of Casework Extraction Buffer was added, and samples were incubated at 70 °C overnight. The day after, 400 μL of lysis buffer was added, and the samples were ready for automated DNA extraction through the Maxwell 16 Instrument. DNA samples were eluted in 50 μL of elution buffer. The Casework Kit is usually used for challenging forensic samples, such as blood stains, semen stains, hair, cigarette butts, tissue samples, and trace DNA samples regularly encountered in forensic DNA analysis. It has also proven to be a good method for extracting DNA from FFPE tissue samples, since it reduces the number of steps and therefore also the risk that the different reagents can further degrade the DNA.

The DNA extraction method used for whole blood samples was performed through the Maxwell^®^ CSC Genomic DNA Kit (catalog number: AS1850, Promega, Madison, WI, USA) by the Maxwell^®^ CSC 48 instrument (Promega). The protocol was followed in all its parts, considering an initial sample volume of 200 µL of whole blood and the volume of elution buffer of 200 µL. DNA extracted from FFPE tissue samples was quantified through a real-time PCR, using the Plexor^®^ HY System kit (catalog number DC1001, Promega, USA). The instructions on the Plexor^®^ HY System Protocol recommend using 2 μL of template DNA per reaction. DNA standards are in the range of 3.2 pg/μL to 50 ng/μL. Performing duplicate analysis of each sample DNA and averaging the quantitation results can reduce variability. Serial dilutions of the DNA standard were amplified in the same run as the unknown samples, and the results were used to generate a standard curve in the autosomal (fluorescein/GREEN) and Y (CALFluor^®^ Orange 560/YELLOW, Berkeley, CA, USA) channels to determine the concentrations of unknown samples. It is necessary to include a no-template control (NTC) reaction for each set of reactions.

For blood samples, quantification was performed using the Qubit dsDNA HS (High Sensitivity) Assay kit (catalog number: Q32851, Thermo Fisher Scientific, Waltham, MA, USA), through the Qubit Instrument.

All the samples were then diluted, reaching a final concentration of 0.67 ng/µL for library preparation with Ion AmpliSeq™ Library Kit 2.0 (catalog number: 4475345, Thermo Fisher Scientific). A volume of 15 µL was aliquoted into the plate for library preparation with the Ion Chef System. The sequencing was then performed through the Ion PGM System for samples 1 and 2, sequenced in 2022 using a custom panel of 97 genes (V1) involved in cardiovascular diseases (18 genes of which were involved in SUDEP according to data collected in the literature). Samples 3, 4, and 5 were recently sequenced through Ion GeneStudio S5 Systems using an updated custom panel with 103 genes (V2), 21 of which are involved in SUDEP. The updated custom gene panel is shown in Supplementary Materials (Table S1). The genes added to the latest version were *DEPDC5*, *DTNA*, *GAA*, *PTPN11*, *SCN1A*, and *SCN8A*. In particular, *DEPDC5*, *SCN1A*, and *SCN8A* are involved in SUDEP. For data analysis, the Torrent Suite (v 5.12.3, TFS), the Integrative Genomics Viewer tool, the Alamut^®^ Visual Plus software (v 1.9), and the Human Gene Mutation Database (HGMD) were used. We validated the NGS variants through Sanger sequencing as a gold-standard technique.

## 3. Results

The extraction methods used with FFPE heart tissue turned out to be quite efficient, since the concentration of DNA was higher than 0.67 ng/µL, as required for library preparation. Results collected by Qubit^®^ and Real-Time PCR by Plexor^®^ HY System are displayed in Table 1.

As expected, very low coverage of the target base was observed for FFPE tissue samples as a result of the complexity of the biological material and the elaborate DNA extraction method. The average base coverage depth and the target base coverage are shown in Table 2. We have adopted the following criteria to discriminate the variants in this study: a total depth at position > 100X for blood samples and >30X for FFPE tissue samples; AF > 0.45 for heterozygous; SNP Strand Bias < 0.6.

Samples were then analyzed and the variants noted through wANNOVAR; subsequently, they were interpreted with the use of bioinformatics tools such as ClinVar, LOVD, CardioClassifier, Revel, and Cadd (Table 3).

All the variants collected in Table 3 are different from each other; none of the samples share the same variants. Therefore, it is very difficult to make assumptions or draw conclusions for a diagnosis of SUDEP, since this does not allow us to define a typical genotype of an SUDEP case.

In particular, in Table 4, we have collected the variants with a relevant clinical significance possibly correlated to the cause of death. In detail, sample 4 resulted in two variants of unknown significance (VUS) on genes *RYR2* and *SCN8A*. Sample 5 resulted in one VUS on the *AKAP9* gene. All three variants are missense, and the variant allele frequency is shown in Table 4. Deepening the molecular significance of these three variants on gnomAD, we concluded that the VUS on the *AKAP9* (NM_005751.5(AKAP9):c.3708A>T) gene is benign, the VUS on *SCN8A* (NM_001330260.2(SCN8A):c.1903C>T) is possibly damaging but predicted as tolerated, and, most interestingly, the VUS on *RYR2* (NM_001035.3(RYR2):c.14302G>A) is indicated as possibly damaging, with consequences on cardiovascular phenotype. All variants were confirmed by Sanger sequencing.

## 4. Discussion

Performing a molecular autopsy on cases of SUDEP was challenging. The main limitation was the small cohort of SUDEP cases due to the rarity of the phenomenon. Hence, to try to broaden the case cohort, we also included in the study FFPE tissue samples from SUDEP cases collected in the past. We have developed a new method of DNA extraction from this complex biological matrix, despite knowing the low coverage in FFPE-derived DNA in NGS analysis. Complying with the results shown in this article, we assume that the choice of FFPE tissue samples prevented a well-conducted genetic analysis. This is due to the presence of formalin, which highly degrades the DNA molecule, leading to its fragmentation and the formation of cross-links. More recently, there has been a growing interest in extracting DNA from FFPE samples for genetic analysis. However, attempting to prepare DNA libraries for NGS from FFPE samples can be particularly challenging due to the effects of formalin fixation and paraffin embedding on the quality of DNA. The DNA extraction method shown in this article was found to be effective, accurate, and fast as a semi-automated method. Some strategies could be worked out from the beginning to overcome some issues, such as following some recommendations on FFPE tissue preparation. In particular, it has been demonstrated that after one day of formalin fixation, the level of DNA fragmentation and formation of cross-links increases in FFPE tissues [11]. Other strategies could be adopted to overcome FFPE tissue problems, such as using optimized pre-treatments like antigen retrieval and deparaffinization, as well as employing specialized extraction kits and technologies to enhance nucleic acid recovery and reduce degradation. The DNA extraction method by the Casework kit, described in this article, allows a reduction in the number of steps and therefore the risk that the different reagents could further degrade the DNA. For these reasons, FFPE tissue samples are usually more prone to errors and false positive variants in NGS analysis. Moreover, false negatives may occur due to the low coverage in FFPE-derived DNA. Therefore, the presence of additional significant variants in non-amplified regions in samples 1, 2, and 3 cannot be excluded. We can infer that for an NGS analysis, it would be better to use blood as a starting biological material in order to have a higher coverage of the target regions. In fact, blood samples 4 and 5 result in a higher coverage analysis. This allowed us to make a more thorough and complete analysis of the whole gene panel, leading to three VUS. The key variants found through NGS analysis and the clinical context of each case are displayed in Supplementary Table S2.

The variant NM_001035.3(RYR2):c.14302G>A in sample 4 was previously discussed in the literature [12,13,14] and classified as a VUS specifically involved in cardiac disease, such as cardiomyopathy [13] and catecholaminergic polymorphic ventricular tachycardia (CPVT) [12,13]. The deceased individual 4 had cardiomyopathy, and this VUS could explain the clinical picture. In particular, the *RYR2* gene encodes the cardiac ryanodine receptor 2 (RyR2), a major component of RyR2 channels, which mediate calcium release from the sarcoplasmic reticulum into the cytosol upon cell membrane depolarization. Defective closure of RyR2 channels results in intracellular calcium leakage, which leads to increased potential for delayed after-depolarizations and subsequent ventricular tachycardia. Mutations in the *RYR2* gene could lead to the most common form of CPVT. As suggested by the literature, *RYR2* mutations could lead to epilepsy and heart disease and are potentially associated with SUDEP [15]. The effect of the variant on protein function is classified as “Uncertain” in the MutationT@ster prediction tool, referring to the fact that the amino acid sequence change might affect the protein features. On Mutation Assessor, this variant has benign support.

The second variant, NM_001330260.2(SCN8A):c.1903C>T, in sample 4, is mentioned in the literature in one case of early infantile epileptic encephalopathy (early-infantile DEE) [14], a neurological disorder characterized by recurring seizures presenting within the first three months of life, progressive cerebral dysfunction, and low electrical brain activity interspersed with bursts of high spiked activity [16]. On MutationT@ster, the variant causes a deleterious protein with an uncertain prediction about the function. On Mutation Assessor, the variant is considered as benign moderate.

The variant NM_005751.5(AKAP9):c.3708A>T, in sample 5, was observed in the literature in Long QT Syndrome and Romano-Ward Syndrome [13], two inherited cardiac diseases that can be correlated with sudden death. Individual 5 was a young woman suffering from epilepsy and in drug treatment and was found lying on the floor of her apartment. Cardiac disease cannot be excluded. Referring to the prediction tools used, MutationT@ster classified the variant as undetermined/benign on the function of the protein, showing an uncertain prediction between different studies in the tool itself.

The three VUS found in this study raise another major problem in genetic analyses. These variants are, in fact, rare and still hold uncertain significance, often because they are related to scarce and poorly studied pathologies or phenomena. These variants need to be interpreted in further studies, since their significance is not certain. Functional tools show different interpretations as well. In order to interpret the correlation between the variant and the pathology in silico, a study is not sufficient; however, it could be suitable to perform experimental in vitro studies, such as RNA-sequencing and functional analysis [17].

## 5. Conclusions

In conclusion, for future studies, we would expand the cohort through multicenter collaborations and integrate genetic data with clinical information to improve interpretation and the genotype-phenotype correlation [18]. To increase the reliability of genetic analyses, we would suggest using the appropriate starting biological material to conduct an NGS analysis, i.e., whole blood rather than FFPE tissue, if available. However, the DNA extraction protocol for FFPE tissue samples used in this study showed good results considering the type of biological matrix. The Casework kit (Promega) is usually used for challenging casework samples, such as in forensic analysis, and it proved adequate for the FFPE samples, too.

Moreover, the NGS custom gene panel may be updated in the future with other genes involved in cardiac disease and SUDEP, detecting additional significant variants if present. Further information on the found VUS in samples 4 and 5 will be provided in the future when functional studies will be conducted, bearing in mind that the interpretation of the found variants requires studies by a multidisciplinary team, including geneticists and clinical and forensic pathologists. It is also essential to perform a careful evaluation of the pathogenicity of the detected gene variants, and the combination of clinical information, pathological examination, and genetic testing through NGS can become a valid approach for SUDEP diagnosis, even in the absence of clinical appearance or typical gross pathology suggesting advanced inherited heart diseases [19].

Although silico instruments predict the possible pathogenicity and classification of some variants, they should not be used as the sole source of evidence to make a clinical assertion [9]. To ascertain the pathogenicity of the variants, studies on larger cohorts will be required. However, collecting a high number of SUDEP cases is a challenge due to the rarity of the event in the population. Molecular autopsy must become an inherent part of the post-mortem examination in cases classified as SUDEP. To date, molecular autopsy is not considered a routine examination, although recommended, which is why many pathologies, such as SUDEP, are underestimated. Moreover, this routine forensic practice should be combined with other clinical evaluations by a neuropathologist or cardiac pathologist for a more comprehensive clinical picture of SUDEP [18,20]. The molecular autopsy not only represents a diagnostic test to ascertain the causes of death of a deceased subject, but is also fundamental for the victim’s family members. Genetic testing could also be performed for family members, who may be at-risk carriers of fatal cardiac disorders. A good interpretation of variants could help to identify the diagnosis or prognosis, preventing sudden death, including the avoidance of medications that may prolong the QT interval, taking antiarrhythmic drugs such as beta-blockers, and prophylactic interventions for possible lethal cardiac arrhythmias, such as implantable cardioverter defibrillators [8]. To best manage families, close interdisciplinary collaboration is essential [9].

## Figures and Tables

**Table 1:** Results of quantification by Qubit^®^ and real-time PCR by Plexor^®^ HY System.

Sample Name	Starting Material	C (ng/µL) by Qubit	C (ng/µL) by Real Time-PCR
Sample 1	10 slices of FFPE		0.7
Sample 2	5 slices of FFPE		1.2
Sample 3	8 slices of FFPE		5.7
Sample 4	200 µL of blood	27.2	
Sample 5	200 µL of blood	18.0	

**Table 2:** This table shows the coverage analysis resulting from the Ion Torrent Suite software. The coverage of the 3 FFPE tissue samples (1, 2, and 3) is very low compared to the coverage of whole blood samples (4 and 5). For the evaluation of variants, we considered 30X as the target base coverage for FFPE tissue-derived DNA and 100X for blood-derived DNA.

Sample Name	% Base Reads on Target	Average Base Coverage Depth	Target Base Coverage at 30X	Target Base Coverage at 100X
Sample 1	38.59%	14.52	7.81%	-
Sample 2	34.59%	6.696	3.47%	-
Sample 3	45.50%	10.63	5.60%	-
Sample 4	95.23%	399.1	99.12%	97.46%
Sample 5	83.49%	271.6	98.76%	93.21%

**Table 3:** Variants found in the epilepsy-related sudden unexpected death cases through NGS analysis, with the updated panel (103 genes).

Sample Name	Gene	Chr	Start	End	Ref	Alt	Clinical Significance *
Sample 3	*MYBPC3*	chr11	47369430	47369430	G	C	Conflicting classifications of pathogenicity **
Sample 4	*ANK2*	chr4	114269433	114269433	A	G	Conflicting classifications of pathogenicity **
	*DSG2*	chr18	29078136	29078136	C	G	Conflicting classifications of pathogenicity **
	*DSP*	chr6	7585967	7585967	G	C	Conflicting classifications of pathogenicity **
	*HCN4*	chr15	73616159	73616159	C	T	Conflicting classifications of pathogenicity **
	*RYR2*	chr1	237972204	237972204	G	A	Uncertain Significance
	*SCN8A*	chr12	52115597	52115597	C	T	Uncertain Significance
Sample 5	*AKAP9*	chr7	91645538	91645538	A	T	Uncertain Significance
	*DSP*	chr6	7585967	7585967	G	C	Conflicting classifications of pathogenicity **

* Bioinformatic tools used for variant interpretation: ClinVar, LOVD, CardioClassifier, Revel, and Cadd (analysis was performed in August 2025). ** Conflicting classifications of pathogenicity refer to situations where the same genetic variant is described differently (e.g., as pathogenic or benign) by different experts or databases, leading to confusion about its potential to cause disease.

**Table 4:** A summary of the results of the analyses performed through IGV (Integrative Genome Viewer) and wANNOVAR. For FFPE tissue samples (1, 2, and 3), no variants of relevant clinical significance were observed. The interpretation of the variants followed the ACMG-AMP guidelines. MutationT@ster 2025 predicted the function of modified proteins.

Sample Name	Gene Region	dbSNP	Population Allele Frequency *	ACMG-AMP Classification	MutationT@ster 2025 Protein Function Prediction	Features of Protein at a Glance
Sample 4	NM_001035.3(RYR2):c.14302G>A p.(Val4768Ile)	rs775534249	0%	VUS: PM1 PM2 PP3	Deleterious	Amino acid sequence changed Protein features (might be) affected
	NM_001330260.2(SCN8A):c.1903C>T p.(Arg635Cys)	rs749983172	0%	VUS: PM2 PP2 BP4	Deleterious	Amino acid sequence changed Protein features (might be) affected
Sample 5	NM_005751.5(AKAP9):c.3708A>T p.(Glu1236Asp)	rs751002727	0.000036%	VUS: BP4 BP1 BP3 PM2	Undetermined/Benign	Amino acid sequence changed Protein features (might be) affected

* Population allele frequency from gnomAD genome.

## Data Availability

The original contributions presented in this study are included in the article/Supplementary Materials. Further inquiries can be directed to the corresponding author.

## Supplementary Materials

The following supporting information can be downloaded at https://www.mdpi.com/article/10.3390/genes16111272/s1: Table S1: The list of genes present in the updated panel used for analysis of samples 3, 4, and 5. A total of 103 genes (V2) were collected; 97 were also present in the past custom gene panel (V1). The genes added to the latest version were *DEPDC5*, *DTNA*, *GAA*, *PTPN11*, *SCN1A*, and *SCN8A*. In particular, *DEPDC5*, *SCN1A*, and *SCN8A* are involved in SUDEP. Table S2: Case Summary Table. It summarizes the most relevant findings (key variants and the clinical significance). V1: Gene Panel version 1 (97 genes), V2: Gene Panel version 2 (103 genes).

## Abbreviations

The following abbreviations are used in this manuscript:

SUDEP	Sudden, Unexpected Death of Someone with Epilepsy
LQTS	Long QT Syndrome
NGS	Next-Generation Sequencing
FFPE	Formalin-Fixed Paraffin-Embedded
VUS	Variant of Unknown Significance
CPVT	Catecholaminergic Polymorphic Ventricular Tachycardia
IGV	Integrative Genome Viewer
Early-Infantile DEE	Early Infantile Epileptic Encephalopathy
