# Supplementary material for: Cardiac Genetic Variants in Sudden, Unexpected Death in Epilepsy: From Challenging DNA Extraction Methods to Updated NGS Panels for Improved Genetic Analysis
Source: Genes (Basel). 2025 Oct 28;16(11):1272. doi: 10.3390/genes16111272 (PMC12652396; doi:10.3390/genes16111272)
Supplement: Supplementary file 1 [file genes-16-01272-s001.zip › genes-3917369-supplementary.pdf]

Custom updated gene panel (V2)

GENE	BrS	LQTS	SQTS	CPVT	HCM	DCM	ARVC	LVNC	ATTR	SUDEP
ABCC9	x					x				
ACTA2										
ACTC1					x	x		x		
ACTN2					x	x		x		
AKAP9	x	x								x
ANK2		x		x						x
ANKRD1						x				
BAG3						x				
CACNA1C	x	x	x		x					
CACNA2D1	x		x							
CACNB2	x		x							
CALM1		x		x						
CALM2	x	x		x						
CALM3		x		x						
CASQ2				x						
CAV3		x								
CDH2							x			
CSRP3					x	x				
CTNNA3							x			
DEPDC5										x
DES					x	x				
DMD						x				
DSC2							x			x
DSG2						x	x			x
DSP							x			x
DTNA								x		
EYA4						x				
FGF12	x									
FHL1					x					
FLNC					x					
GAA										
GATA6										
GLA					x					
GPD1L	x									
HCN4	x							x		x
HEY2	x									
JPH2					x					
JUP							x			x
KCND2	x									
KCND3	x									
KCNE1		x								x
KCNE2		x								x
KCNE3	x									
KCNE5	x									
KCNH2	x	x	x							x
KCNJ2		x	x	x						
KCNJ5		x								

KCNJ8	x									
KCNQ1		x	x							x
LAMP2					x					
LDB3						x	x	x		x
LMNA						x	x	x		
MYBPC3					x	x		x		x
MYH6					x	x				x
MYH7					x	x		x		
MYL2					x					
MYL3					x					
MYLK										
MYLK2					x	x				
MYOZ2					x					
MYPN					x	x				
NEXN					x	x				
NKX2-6										
NOS1AP		x								
PKP2					x	x	x			
PLN	x				x		x	x		
PRKAG2					x					
PSEN2						x				
PTPN11					x					
RAF1					x					
RANGRF	x									
RBM20						x				
RIT1					x					
RYR2		x		x			x	x		x
SCN10A	x									x
SCN1A										x
SCN1B	x	x								x
SCN2B	x									
SCN3B	x									
SCN4B		x								
SCN5A	x	x				x				x
SCN8A										x
SEMA3A	x									
SGCD						x				
SLC8A1							x			
SLMAP	x									
SNTA1		x								
TAZ						x		x		
TCAP					x	x				
TECRL	x			x						
TGFB2										
TGFB3							x			
TMEM43							x			
TMPO						x				
TNNC1					x	x				
TNNI3					x	x				
TNNT2					x	x		x		
TPM1					x	x		x		

TRDN		x		x						
TRPM4	x									
TTN					x	x	x			
TTR					x				x	
VCL						x				

Table S1: It shows the list of genes present in the updated panel used for analysis of samples 3, 4 and 5. 103 genes (V2) are collected in the tables, 97 were present also in the past custom gene panel (V1). The genes added to the latest version were DEPDC5, DTNA, GAA, PTPN11, SCN1A, SCN8A. In particular, DEPDC5, SCN1A and SCN8A are involved in SUDEP.

BrS: Brugada Syndrome
LQTS: Long QT Syndrome
SQTS: Short QT Syndrome
CPVT: Catecholaminergic polymorphic ventricular tachycardia
HCM: Hypertrophic cardiomyopathy
DCM: Dilated cardiomyopathy
ARVC: Arrhythmogenic right ventricular cardiomyopathy
LVNC: Left ventricular noncompaction cardiomyopathy
ATTR: Transthyretin amyloid cardiomyopathy (ATTR-CM)
SUDEP: Sudden and Unexpected death in Epilepsy

Sample Name	Clinical Context	DNA extraction kit	Sequencing Platform	Gene Panel	Key Variants
**Sample 1 FFPE**	Definite SUDEP	Casework Kit	Ion PGM	V1	
**Sample 2 FFPE**	Definite SUDEP	Casework Kit	Ion PGM	V1	
**Sample 3 FFPE**	Definite SUDEP	Casework Kit	Ion GeneStudio S5	V2	
**Sample 4 Whole blood**	Definite Plus SUDEP	CSC Genomic DNA Kit	Ion GeneStudio S5	V2	**NM_001035.3(RYR2):c.14302G>A p.(Val4768Ile)**<br>**NM_001330260.2(SCN8):c.1903C>T p.(Arg635Cys)**
**Sample 5 Whole blood**	Definite SUDEP	CSC Genomic DNA Kit	Ion GeneStudio S5	V2	**NM_005751.5(AKAP9):c.3708A>T p.(Glu1236Asp)**

Table S2: Case Summary Table. It summerizes the most relevant findings (key variants and the clinical significance).
V1: Gene Panel version 1 (97 genes)
V2: Gene Panel version 2 (103 genes)
